# Oxytocin Reduces the Attractiveness of Silver-Tongued Men for Women During Mid-Cycle

**DOI:** 10.3389/fnins.2022.760695

**Published:** 2022-04-28

**Authors:** Zhao Gao, Xiaole Ma, Xinqi Zhou, Fei Xin, Shan Gao, Juan Kou, Benjamin Becker, Keith M. Kendrick

**Affiliations:** ^1^The Clinical Hospital of Chengdu Brain Science Institute, University of Electronic Science and Technology of China, Chengdu, China; ^2^Key Laboratory for Neuroinformation, Ministry of Education, School of Life Sciences and Technology, University of Electronic Science and Technology of China, Chengdu, China; ^3^School of Foreign Languages, University of Electronic Science and Technology of China, Chengdu, China; ^4^School of Educational Science, Shanxi University, Taiyuan, China; ^5^Institute of Brain and Psychological Sciences, Sichuan Normal University, Chengdu, China

**Keywords:** oxytocin, menstrual cycle, metaphor, attractiveness, mate choice

## Abstract

In humans, the neuropeptide oxytocin promotes both attraction toward and bonds with romantic partners, although no studies have investigated whether this extends to the perceived attractiveness of flirtatious language. In a within-subject, randomized double-blind placebo-controlled behavior and functional magnetic resonance imaging (fMRI) paradigm (https://clinicaltrials.gov/show/NCT03144115), 75 women rated the attractiveness of either a male face alone or paired with a verbal compliment which varied in terms of topic (women or landscapes) and figurativeness (novel or conventional metaphors or literal expressions). Subjects were tested in fertile and luteal phases of their cycle and on both occasions received either 24 IU intranasal oxytocin or placebo. Results showed that, whereas under placebo women in the fertile phase rated the facial attractiveness of men producing novel metaphorical compliments higher than in their luteal phase, following oxytocin treatment they did not. Correspondingly, under oxytocin the faces of individuals producing novel metaphorical compliments evoked greater responses in brain regions involved in processing language (middle frontal gyrus) and cognitive and emotional conflict (posterior middle cingulate and dorsal anterior cingulate) but reduced functional connectivity between the dorsal anterior cingulate and right orbitofrontal and medial frontal gyri. Thus, sex hormones and oxytocin may have opposite effects in regulating mate selection in women during their fertile phase. Novel metaphorical compliments convey a greater sexual than bonding intention and thus while sex hormones at mid-cycle may promote attraction to individuals communicating sexual rather than bonding intent, oxytocin may bias attraction away from such individuals through increasing cognitive and emotional conflict responses toward them.

## Introduction

In line with evolutionary aesthetics, language preference that is rooted in pleasant and rewarding emotions may have influenced its subsequent evolution (Rolls, 2017). Notably, some language attributes, such as novel metaphors in emotional contexts (Yang et al., 2019), may have had particular evolutionary significance in this respect (Miller, 2000; Jablonka et al., 2012). As the author Jane Hirshfield has observed: “Metaphors get under your skin by ghosting right past the logical mind.”. Indeed, metaphor preference is neurobiologically underpinned to attract us similarly to physical attributes (Rolls, 2017). For example, cognitive processing of metaphors has been well documented to rely on different neural networks from that of literal expressions (Rapp et al., 2012; Diaz and Eppes, 2018) due to the degree of novelty (Schmidt and Seger, 2009). In a recent study (Gao et al., 2017a,b), we have also found that women rate men who used metaphorical compliments to praise them as more attractive than ones using literal expressions. The perceived courtship motive was also found to vary with the topic or linguistic figurativeness of men’s compliments (Bale et al., 2006; Hall et al., 2008; Hendon, 2012; Gao et al., 2017b).

Relatively little is known about whether there are similar influences of hormones on the perceived attractiveness of different figurativeness of courtship language used by men similar to their well-established effects on a number of non-verbal features (de Boer et al., 2012; Gangestad and Grebe, 2017; Jones et al., 2019). Fluctuations in sex hormones during the menstrual cycle have been shown to influence non-semantic aspects of language such as voice pitch (Puts, 2005). Only one study has reported that at mid-cycle, women pay greater attention to hearing language expressing overt courtship in a dichotic listening task (Rosen and López, 2009) and that this attentional bias shows some association with estrogen levels. In a previous study, we also found that women in an existing relationship preferred men using verbal compliments praising their appearance during their fertile phase but that single women showed this preference in their luteal phase. However, no interaction was found between the menstrual cycle and the figurativeness of the compliments used (Gao et al., 2017a). The measurement of the fertility window used in this previous study was only based on self-reported menstrual cycle information and could be improved using direct hormone measures, namely concentrations of luteinizing hormone (LH – Marcinkowska et al., 2016). Thus, currently, the extent to which the attractiveness of different figurativeness of courtship language may be influenced by cycle-dependent hormonal changes is unclear.

Another hormone that may potentially influence the attractiveness of different figurativeness of courtship language is the hypothalamic neuropeptide oxytocin (OT). This peptide has been referred to as a candidate “love hormone” (Carter and Perkeybile, 2018), although it has many different reported effects on social cognition in both animal models and humans (Striepens et al., 2011; Kendrick et al., 2017). In animal models, oxytocin release plays an essential role in promoting the formation and maintenance of both parental and pair bonds primarily by enhancing the impact of relevant sensory cues such as smell, sight, and touch on neural systems involved in processing them as well as in salience and reward networks (Kendrick, 2000; Carter and Perkeybile, 2018). In humans, oxytocin administration has also been shown to increase the attractiveness of visual and auditory social cues and responses to social, affective touch primarily by increasing their impact on salience and reward brain networks (Groppe et al., 2013; Scheele et al., 2013; Striepens et al., 2014; Hu et al., 2015; Yao et al., 2018; Chen et al., 2020a,b). Thus, for example, oxytocin administration increases the perceived attractiveness of the faces of both male and female partners in an existing relationship and correspondingly increases responses to them in brain reward regions (Scheele et al., 2013, 2016). However, while it can increase the attractiveness of perceived sensory cues when these are combined with information in the form of verbal descriptors about an individual, it can also reduce it. For example, in women, while oxytocin increases the attractiveness of the faces of men who have been faithful in a previous relationship, it has the opposite effect for men who have been unfaithful (Xu et al., 2020). There is also increasing evidence from these paradigms that oxytocin tends to amplify sex differences in terms of what characteristics men and women consider attractive in others (Gao et al., 2016; Xu et al., 2020). Finally, oxytocin has also been shown to help maintain romantic bonds between couples by reducing couple conflict (Ditzen et al., 2009), jealousy in couples evoked by either imagined or simulated partner infidelity (Zheng et al., 2021), and for men in a relationship showing reduced motivation for approaching attractive female strangers (Scheele et al., 2012). However, none of these studies has examined whether oxytocin influences the attractiveness of the type of language used by others, although one study has reported that it enhances recognition of positive sexual and relationship words (Unkelbach et al., 2008), suggesting that it may influence responsivity to courtship language.

We, therefore, aimed to explore in a double-blind, randomized placebo-controlled pharmaco-fMRI study whether intranasal OT influences behavioral and neural responses of women in either their fertile or luteal phases to different topics (appearance or landscape) and figurativeness (literal and conventional or novel metaphor) of verbal compliments paid by unfamiliar men. In addition to the self-report method for the estimation of fertile phase (Gangestad et al., 2016), urinary LH detection strips were used to more accurately identify the ovulation window. Based on previous studies, we hypothesized that OT would enhance the attractiveness of the faces of men producing metaphorical compliments targeting appearance during the fertile phase and that this would be associated with enhanced responses in both salience and reward processing networks.

## Materials and Methods

### Participants

Seventy-five healthy young women (*M*_age_ ± SD = 19.68 years ± 1.53) from the University of Electronic Science and Technology of China were recruited to participate in two identical experiments twice – once in their fertile phase and once in their luteal phase. Recruitment criteria included: (1) being single > 3 months, (2) having a regular menstrual cycle, (3) no intake of oral contraceptives, and (4) no medical history of mental or neurological disorders. Thirteen participants were excluded due to development of illness (*n* = 4) or change in romantic relationship status (*n* = 3) between the assessments or due to excessive movement during either of the fMRI sessions (*n* = 6), leaving a total of *n* = 62 participants (*N*_OT_ = 31, *N*_PLC_ = 31) for the analysis. Although the original number of recruited subjects (*n* = 75) achieved >90% power (G*Power) for main and interaction effects in mixed ANOVAs with an expected medium effect size (partial eta squared of 0.06), the power still reaches 80% for 62 subjects who were included into the final analysis. All subjects attended three visits. During the first visit scheduled 1 week before the first of the two experimental sessions, a series of questionnaires were completed to control for potential between-group differences (see Table 1): (1) demographic information, including age, menstrual cycle, and love experiences; (2) romantic love – Passionate Love Scale (PLS; Hatfield and Sprecher, 1986); and (3) emotional traits that may influence the experimental assessments, including Cheek and Buss Shyness Scale (CBSS; Cheek and Buss, 1981), Self Esteem Scale (SES; Rosenberg, 1965), State Trait Anxiety Inventory (STAI; Wenli and Mingyi, 1995); Beck Depression Inventory (BDI; Beck et al., 1996), and the Empathy Question (EQ; Mehrabian and Epstein, 1972).

**TABLE 1:** Demographic info and psychological measurements of participants.

	OT group (*N* = 31)		PLC group (*N* = 31)		*t*	*p*
	Mean	*SE*	Mean	*SE*		
Age (year)	20.06	0.36	19.30	0.19	1.89	0.06
Menstrual cycle (day)	28.93	0.53	29.86	0.71	−1.05	0.30
EQ	39.77	2.10	43.00	1.69	−1.20	0.23
BDI	8.30	1.31	7.74	1.04	0.33	0.74
CBSS	35.74	1.70	34.52	1.46	0.55	0.59
SES	31.52	0.81	31.32	0.61	0.19	0.85
TAI	42.26	1.51	42.65	1.31	−0.19	0.85
SAI (fertile phase)	39.59	1.77	38.00	1.22	0.75	0.46
SAI (luteal phase)	36.68	1.75	38.40	1.71	−0.70	0.49
PLS	84.45	3.87	89.84	3.30	−1.06	0.29

**FIGURE 1:**
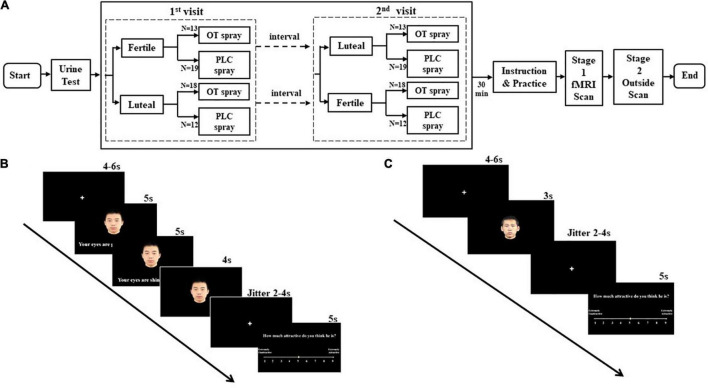
Experiment design. **(A)** Flow chart of study protocol. **(B)** A trial of a face paired with a compliment using a conventional metaphor targeting appearance condition (“face + CMA”) during fMRI. **(C)** A trial presenting a face alone (“face alone” condition) during fMRI.

The experiment was a double-blind placebo-controlled within-subject pharmacological fMRI protocol during which participants received either OT or placebo during two study visits. Specifically, 18 out of 31 women in OT treatment group participated study firstly during their luteal phase and secondly during their fertile phase while 13 women in the same group firstly during fertile phase and secondly during luteal phase (Figure 1A; for experiment design). The order effect and interval time were controlled (see Table 2). Each participant received a nasal spray containing OT (24 IU; Sichuan Meike Pharmaceutical Co. Ltd., Sichuan, China; the People’s Republic of China GMP No. SC20140046, OT) or a placebo (PLC) spray containing the same ingredients except for the neuropeptide and provided in identical bottles. Within each participant, treatment was held constant during the two experimental sessions. Administration procedures strictly followed a standard protocol (Guastella et al., 2013), with three puffs of 4 IU administered per nostril at 30-s intervals. In order to control for treatment effects on mood, the Positive and Negative Affect Schedule (PANAS - Lin et al., 2008) was administered before and after treatment, and data analysis revealed no significant difference between treatment groups (*t* = 0.75, *p* = 0.46). To determine the ovulatory phase of the menstrual cycle, we used urinary LH detection strips (Jin Xiuer©; Guangzhou Wondfo Biotechnology Co. Ltd., Guangzhou China) in combination with self-reported onset time and duration of cycles during the previous 3 months. Each participant received training on the last day of menses and began to self-administer urine tests 5 days before the estimated date of ovulation. Results were recorded and reported daily until the LH value reached 30 miu/ml or above, at which point subjects participated in the fertility phase pharmacological fMRI experiment within 36 h. Five days before the estimated onset day of menses, participants repeated the self-test protocol and, when their urinary LH value was lower than 20 miu/ml, underwent the luteal phase of the experiment within 36 h. For confirmation, all the participants received the urinary LH test again immediately before the beginning of the experiment.

**TABLE 2:** Experiment protocol of order^a^ and treatment group on the interval time between two visits and the number of participants.

	OT group (*N* = 31)			PLC group (*N* = 31)			Interval time		Number^b^
	*N*	Mean days	*SE*	*N*	Mean days	*SE*	Group	Order	
Order A. *1L-2F*	18	28.67	12.31	12	36.42	15.61	*F* = 0.010	*F* = 2.821	χ^2^ = 2.325
Order B. *1F-2L*	13	29.38	20.64	19	22.42	13.20	*p* = 0.921	*p* = 0.098	*p* = 0.204

*^a^Women participants completed two equivalent experimental sessions once during their fertile phase and once during their luteal phase, i.e., Order A. 1L-2F: the first experiment in luteal phase and the second experiment in fertile phase, and Order B. 1F-2L: the first experiment in fertile phase and the second experiment in luteal phase. Interval time refers to days between two experiments.*

*^b^Chi-square crosstab test (2 treatment group × 2 order) on the number of participants. Fisher’s exact test, two-sided exact significance at the level of p < 0.05.*

Experimental procedures were approved by the local Ethics Committee of the University of Electronic Science and Technology of China, in accordance with the latest revision of the Declaration of Helsinki, and pre-registered on the clinicaltrials.gov repository (ID: NCT03144115^1^). Data were collected between October 2016 and July 2018. Every participant provided written informed consent and received monetary compensation (RMB135 per experimental session). Initially, we planned to recruit 80 subjects. However, due to a technical upgrade of the fMRI system, only 75 subjects were enrolled in the study.

### Language Stimuli

Sixty-nine male participants recruited in a prior study (Gao et al., 2017a) had produced 750 sentences to compliment five different aspects of either the appearance of women (face, eyes, lips, hair, or smile) or natural landscapes (mountains, sky, shade, cloud, and lake). All the sentences were categorized into three types of figurativeness (novel metaphor, conventional metaphor, and literal expression) and two topics (appearance or landscape), thus forming 6 types of compliments: novel metaphor targeting appearance (NMA), conventional metaphor targeting appearance (CMA), literal expression targeting appearance (LEA), novel metaphor targeting landscape (NML), conventional metaphor targeting landscape (CML) and literal expression targeting landscape (LEL). According to CCL Corpus provided by the Center for Chinese Linguistics at Peking University^2^, each complimentary sentence was matched across all conditions for length (average length = 11.2 characters, range: 9–15 characters) and word frequency (mean = 435199.4, *SD* = 176226.2). An independent group of 55 female participants (*M*_age_ = 20.02 years, *SD* = 2.71) was recruited to rate sentences regarding appropriateness, valence, familiarity, figurativeness, arousal, and imageability on 7-point Likert scales (1 = lowest, 7 = highest). Sentences rated on average higher than 4 in figurativeness but lower than 4 in familiarity were stratified into the novel metaphor category, whereas those rated above 4 in both figurativeness and familiarity were included in the conventional metaphor category. Compliments, the figurativeness rating of which was below 3, were categorized as literal expressions. For example, “*Your face is a legendary picture.*” or “*Your smile is a hot summer*.” belong to novel metaphors targeting appearance; “*Mountains in the distance are a sleeping giant.*” or “*The sky today is a blue dome.*” belong to conventional metaphors targeting landscape; “*Your hair is so smooth.*” or “*The cloud at the edge of the sky is so white.*” belong to literal expressions. Finally, 288 sentences selected for the experiment all conformed with appropriateness for use in the Chinese language (*M*_appropriateness_ > 4). One-factor analysis of variance (ANOVA) and *post hoc* Bonferroni corrected tests were used to examine group differences in the ratings (see Supplementary Table 1). The 288 experimental sentences were then balanced across the 6 criteria and assigned randomly to be paired with 144 different neutral expression male faces. Every face was paired with 2 compliments of the same category. As a control condition, an additional 15 faces were not paired with any sentences.

In order to confirm the perceived intention of novel metaphorical compliments used by unfamiliar men in a between-sex communication, an independent group of 42 female participants was recruited to rate all the novel metaphorical compliments in a mating context. A paired-sample *t*-test showed that female participants rated such compliments as indicating a significantly greater sexual and short-term relationship interest (flirting) than a bonding and long-term relationship interest (*M*_Proportion of flirting_ ± SE = 55.41% ± 1.72, *M*_Proportion of bonding_ ± SE = 44.50% ± 1.73, *t* = 3.163, *p* = 0.003).

### Face Stimuli

Five hundred male face photos were acquired with permission from each individual when male undergraduates signed up for the National College English Test. Photoshop CS6.0 (Adobe System Inc.) was used for photo standardization: to remove hair, cover clothes, and change the background color into black; to unify color contrast, brightness, pixel number, and size. Thus, unspecific visual features of the pictures that might influence attractiveness ratings were controlled.

On a 9-point Likert scale, an independent group of 19 women (*M*_age_ = 19.51 years, *SD* = 0.88) rated face attractiveness (1 = very unattractive, 9 = very attractive), valence (1 = very negative, 9 = very positive) and trustworthiness (1 = strongly distrust, 9 = strongly trust). Finally, 318 neutral faces with medium attractiveness ratings between 3.5 and 4.5 (*M*_attractiveness_ ± SE = 4.13 ± 0.02, *M*_valence_ ± SE = 4.95 ± 0.02, *M*_trustworhiness_ ± SE = 4.57 ± 0.01) were randomly assigned into the ‘face + compliment,’ ‘face alone’ control condition, and ‘new face’ condition for a subsequent memory test including the previous presented and novel faces (Gao et al., 2017a). There were no significant differences between the stimulus sets in terms of the rated characteristics (*M*_attractiveness_ ± SE = 4.11 ± 0.03 ∼ 4.18 ± 0.06, *F*_5_,_318_ = 0.239, *p* = 0.954; *M*_valence_ ± SE = 4.88 ± 0.07 ∼ 4.99 ± 0.03, *F*_5_,_318_ = 0.673, *p* = 0.644; *M*_trustworthiness_ ± SE = 4.53 ± 0.08 ∼ 4.59 ± 0.03, *F*_5_,_318_ = 0.331, *p* = 0.894). A total of 159 facial stimuli were used for the fMRI paradigm and randomly paired with written compliments (i.e., 6 categories of compliments, 12 compliments/category^•^session) or none (15 faces/session).

### Procedure

Women participants completed two equivalent experimental sessions once during their fertile phase and once during their luteal phase. See Figure 1A for more details.

An LH urine test was first administered on each experiment day, followed immediately by intranasal spray administration. Starting 30 min after the intranasal spray was administered, the experiment was introduced to the participants stating that the complimentary sentences were derived from poetry or prose composed by the male writers to compliment their future partners. Women participants then practiced the attractiveness rating task with five novel female faces. At 45 min after treatment administration, the within-scanner stage of the experiment began in which stimuli were presented in a mixed design and distributed over 3 runs (balanced for compliment topic and figurativeness) with 87 trials per run (duration per run: 730 s). Stimuli conditions were face alone, face + NMA, face + CMA, face + LEA, face + NML, face + CML, and face + LEL. In total, there were 12 trials in each of the conditions other than for the face-alone condition where there were 15 trials. In all the conditions of face plus sentences, every face was paired with 2 different compliments of the same category, and each sentence was presented for 5 s. When the second sentence disappeared after a jitter of 5–7 s, subjects rated the male’s attractiveness on a 9-point scale (1 = strongly unattractive, 9 = strongly attractive) (see Figure 1B). In the face-alone condition, the male’s attractiveness was rated after a 3-s face presentation (see Figure 1C).

In the outside-scanner stage, ratings of faces were given again but without sentences, and a surprise recognition test was administered. A pool of 159 faces without paired sentences was created, which comprised 87 faces that had been used in the previous stage and 72 novel faces. Each face was then presented in randomized order on the computer screen for 3 s, and then within 20 s, subjects rated the attractiveness of each male face alone on a 9-point scale and reported whether they had seen them in the previous part of the experiment (yes or no = forced-choice recognition memory test).

### Data Analysis

#### Behavioral Data Analysis

A mixed ANOVA with 2 compliment topics (women appearance vs. landscape) × 3 compliment figurativeness (literal expression vs. conventional metaphor vs. novel metaphor) × 2 menstrual phases (fertile vs. luteal) as within-subject factors and treatment group (OT vs. PLC) as a between-subject factor was performed using SPSS 25.0. Paired-sample *t*-tests were used for either within each treatment group or all subjects to confirm effects of language stimuli and exclude familiarity effects of face stimuli: (1) compared the ratings of faces that had not been paired with language between two stages; (2) compared the ratings in the in-scanner stage between the conditions of “face + compliment” and “face alone,” and (3) compared the ratings of faces in the outside-scanner stage between those that had been paired with language in the previous stage and those new faces in this stage. In this first paper, we only focus on the behavioral data collected during the fMRI scans. Multiple comparisons were all Bonferroni corrected with *p* < 0.05 considered to be significant.

#### Acquisition and Analysis of Functional Magnetic Resonance Imaging Data

Functional MRI data were obtained on a 3T GE Discovery MR750 system (General Electric, Milwaukee, WI, United States) employing blood oxygenation level-dependent (BOLD) imaging. Echo-planar images were acquired with a gradient echo-planar imaging sequence (TR, 2,000 ms; TE, 30 ms; slices, 43; thickness, 3.2 mm; gap, 0 mm; FOV, 220 mm × 220 mm; flip angle, 90°; matrix size, 64 × 64; voxel size, 3.4 mm × 3.4 mm × 3.2 mm). High-resolution whole-brain volume T1-weighted images were acquired (Spoiled gradient echo pulse sequence; TR, 5.92 ms; TE, 1.956 ms; flip angle, 12°; FOV, 256 mm × 256 mm; thickness, 1 mm; matrix, 256 × 256 × 176; voxel size, 1 mm × 1 mm × 1 mm) to identify subjects with apparent anatomical abnormalities and increase normalization accuracy during fMRI data preprocessing.

Imaging data were processed with SPM 12 (Statistical Parametric Mapping; Wellcome Department of Cognitive Neurology, London, United Kingdom^3^) implemented in Matlab (R2014a, MathWorks, Inc., Natick, MA, United States) (Friston et al., 1994). The first five volumes of each functional time series were discarded to allow for T1 equilibration. Images were corrected for head movement between scans by an affine registration. A two-pass procedure was used by which images were initially realigned to the first image of the time series and subsequently realigned to the mean of all images. For spatial normalization, the mean T1 image of each subject was normalized to the current Montreal Neurological Institute (MNI) template. All functional images were hereby transformed into standard MNI space and resampled at 3 mm × 3 mm × 3 mm voxel size. The normalized images were spatially smoothed using an 8-mm FWHM Gaussian kernel.

The 1st-level data matrix included the following experimental conditions separately for both fertile and luteal phase: faces + compliments (NMA, NML, CMA, CML, LEA, and LEL), faces presented alone that had been paired with compliments, faces presented alone that had never been paired with compliments as well as the rating period and 6 head movement parameters as a covariate to improve motion control. Given that the study’s main aim was to explore whether OT’s modulatory effect on metaphoric preference fluctuates across the menstrual cycle, the second level group analysis primarily focused on the interaction effect between drug, complimentary figurativeness, and menstrual cycle. Therefore, an ANOVA implemented in a flexible factorial design with the factors 2 treatments (OT vs. placebo) by 3 figurativeness (novel metaphor vs. conventional metaphor vs. literal expression) by 2 cycle phases (fertile vs. luteal) was first established to determine the interaction effect. Based on the findings from the behavioral data, the following 2-way factorial models of 2 treatments (OT vs. placebo) by 2 cycle phases (fertile vs. luteal) were employed, focusing on novel metaphors, conventional metaphors, and literal expressions. *Post hoc* independent-sample *t*-tests were further conducted to determine group differences. All analyses used a whole-brain approach with a significance threshold of *p* < 0.05 corrected for multiple comparisons at the cluster level with an initial cluster forming threshold of *p* < 0.001 and Family-wise error (FWE) correction. Finally, a Spearman correlation was conducted to examine associations between attractiveness ratings and extracted beta estimates of brain activity at a significance level of 0.05.

In accordance with the findings from the analysis of neural activation, treatment effects on the task-modulated functional connectivity of the identified regions were further examined using gPPI (Generalized Form of Context-Dependent Psychophysiological Interactions) (McLaren et al., 2012).

## Results

### Behavioral Results

**FIGURE 2:**
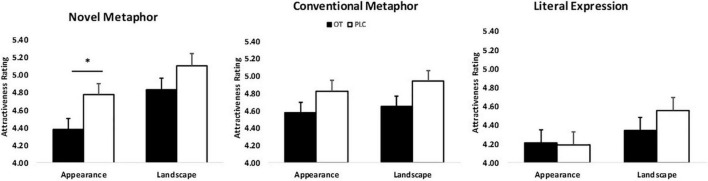
Topic × figurativeness of men’s compliments × treatment interaction affects attractiveness ratings (*n* = 62). *p < 0.05, two-tailed *t*-test. Bars indicate *M* ± SE.

The results of the mixed ANOVA revealed a main effect of figurativeness (*F*_2_,_120_ = 51.72, *p* < 0.001, $\eta_{\text{p}}^{2}$ = 1.00) and topic (*F*_1_,_60_ = 31.04, *p* < 0.001, $\eta_{\text{p}}^{2}$ = 1.00) on attractiveness ratings. Ratings were significantly higher for men whose faces were paired with either metaphorical compliments (both novel and conventional) relative to literal expressions (Mean ± SE: NM = 4.77 ± 0.09, CM = 4.75 ± 0.08, LE = 4.32 ± 0.09; *p*s < 0.001) or any figurativeness of compliments targeting landscape relative to those targeting women’s appearance (Mean ± SE: Landscape = 4.49 ± 0.09, Appearance = 4.73 ± 0.09; *p* < 0.001). Moreover, a two-way interaction between topic and figurativeness was found (*F*_2_,_120_ = 11.42, *p* < 0.001, $\eta_{\text{p}}^{2}$ = 0.99) reflecting that men whose faces were paired with compliments in the form of novel metaphors or literal expressions targeting women’s appearance were both rated less attractive than those targeting landscape (Mean ± SE: NM_appearance_ = 4.57 ± 0.09, NM_landscape_ = 4.96 ± 0.10, LE_appearance_ = 4.20 ± 0.10, LE_landscape_ = 4.45 ± 0.10; *p*s < 0.001) whereas no significant difference between topics was found for the compliments using conventional metaphors (Mean ± SE: CM_appearance_ = 4.70 ± 0.09, CM_landscape_ = 4.79 ± 0.09, *p* = 0.06) (see Supplementary Figure 1). The interaction between figurativeness and treatment was also significant (*F*_2_,_120_ = 3.16, *p* = 0.046, $\eta_{\text{p}}^{2}$ = 0.60). Additionally, there was a significant figurativeness * topic * treatment interaction (*F*_2_,_120_ = 4.07, *p* = 0.02, $\eta_{\text{p}}^{2}$ = 0.71). *Post hoc* Bonferroni corrected tests revealed that OT, relative to PLC, decreased attractiveness ratings of men who used novel metaphors to pay compliments for women’s appearance (Mean ± SE: NM_OT_ = 4.38 ± 0.13, NM_PLC_ = 4.77 ± 0.13; *p* = 0.032) (see Figure 2).

**FIGURE 3:**
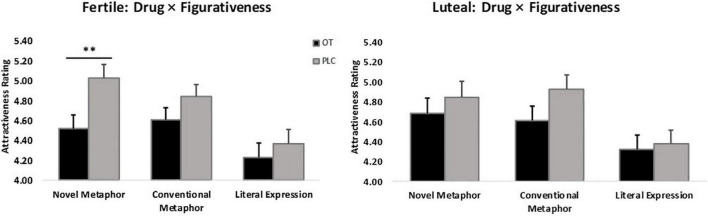
Women’s menstrual cycle × figurativeness of men’s compliments × treatment interaction affects attractiveness ratings (*n* = 62). **p < 0.01, two-tailed *t*-test. Bars indicate *M* ± SE.

**FIGURE 4:**
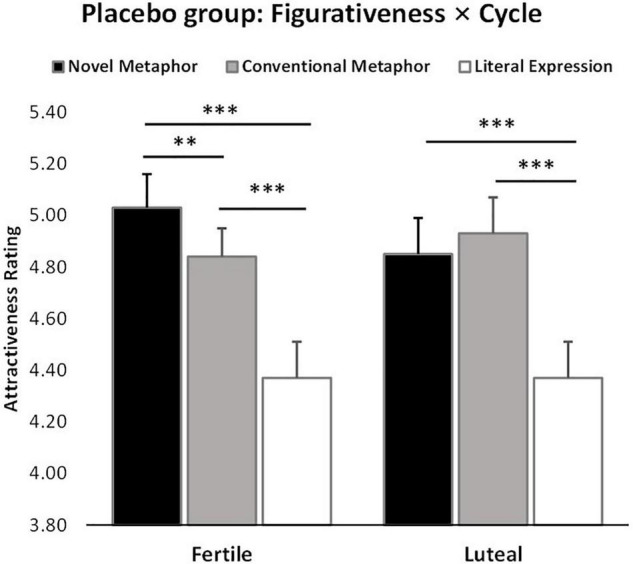
Cycle × figurativeness interaction effect on attractiveness ratings independent of treatment (*n* = 62). ^**^p < 0.01, ^***^p < 0.001, two-tailed *t-*test. Bars indicate *M* ± SE.

There was also a significant figurativeness * menstrual cycle * treatment interaction (*F*_2_,_120_ = 4.51, *p* = 0.01, $\eta_{\text{p}}^{2}$ = 0.76), in line with the main research focus of the study. *Post hoc* Bonferroni corrected tests showed that women in their ovulatory phase in the OT treatment group gave significantly lower attractiveness ratings to men who used novel metaphorical compliments than those in the PLC group (Mean ± SE – fertile: NM_OT_ = 4.52 ± 0.13, NM_PLC_ = 5.03 ± 0.13, *p* = 0.01). No other group differences reached statistical significance in the other conditions (Mean ± SE – fertile: CM_OT_ = 4.61 ± 0.12, CM_PLC_ = 4.84 ± 0.12; LE_OT_ = 4.23 ± 0.14, LE_PLC_ = 4.37 ± 0.14; luteal: NM_OT_ = 4.68 ± 0.16, NM_PLC_ = 4.85 ± 0.16; CM_OT_ = 4.61 ± 0.14, CM_PLC_ = 4.93 ± 0.14; LE_OT_ = 4.32 ± 0.14, LE_PLC_ = 4.38 ± 0.14; *p*s > 0.1), see Figure 3. Further analysis of the two-way interaction between figurativeness and menstrual cycle within each treatment group showed a main effect of figurativeness in both groups (OT: *F*_2_,_60_ = 21.95, *p* < 0.001, $\eta_{\text{p}}^{2}$ = 1.000; PLC: *F*_2_,_60_ = 30.27, *p* < 0.001, $\eta_{\text{p}}^{2}$ = 1.000), but an interaction only in the PLC group (Mean ± SE: NM_fertile_ = 5.03 ± 0.13, CM_fertile_ = 4.84 ± 0.11, LE_fertile_ = 4.37 ± 0.14, NM_luteal_ = 4.85 ± 0.14, CM_luteal_ = 4.93 ± 0.14, LE_luteal_ = 4.38 ± 0.14), *F*_2_,_60_ = 4.04, *p* = 0.02, $\eta_{\text{p}}^{2}$ = 0.70, see Figure 4, suggesting that the faces paired with novel metaphors were rated significantly more attractive than those paired with conventional metaphors and literal expressions independent of treatment. In order to clarify the modulatory effect of both cycle and OT, the separate ANOVA analysis for each condition of compliment figurativeness revealed a non-significant treatment x cycle interaction in the novel metaphor condition (*F*_1_,_60_ = 2.99, *p* = 0.089, $\eta_{\text{p}}^{2}$ = 0.40). However, an exploratory *post hoc* analyses using Bonferroni corrected *t*-tests revealed a significant effect of OT for novel metaphors in the fertile condition (*t* = −2.704, *p* = 0.009 – Mean ± SE: OT_NM–fertile_ = 4.52 ± 0.13, PLC_NM–fertile_ = 5.03 ± 0.13) but not in the luteal phases (*t* = −0.751, *p* = 0.456; OT_NM–luteal_ = 4.68 ± 0.16, PLC_NM–luteal_ = 4.85 ± 0.16). No significant interaction between cycle and treatment was found in the other two compliment conditions (see Supplementary Figure 2).

Paired-sample *t*-test analysis showed that during the stage of fMRI acquisition, the attractiveness ratings for men in the condition of “face + compliment” were significantly higher than in the “face alone” condition [Mean ± SE – “face + compliment” = 4.61 ± 0.08, “face alone” = 4.03 ± 0.09, *t*(62) = 10.31, *p* < 0.001]. In the subsequent post-fMRI rating session, the attractiveness ratings of faces that had been paired with compliments in the first stage significantly decreased [Mean ± SE – Stage 1 = 4.61 ± 0.08, Stage 2 = 3.80 ± 0.11, *t*(61) = 12.18, *p* < 0.001], as did ratings in the “face alone” condition [Mean ± SE – Stage 1 = 4.06 ± 0.09, Stage 2 = 3.66 ± 0.11, *t*(61) = 6.30, *p* < 0.001]. However, the faces of men which had been paired with compliments in the fMRI acquisition stage were rated significantly more attractive than novel men’s faces included as familiarity control stimuli [Mean ± SE: faces “paired with compliments in stage 1” = 3.80 ± 0.11, faces “added newly” = 3.67 ± 0.11, *t*(61) = 7.63, *p* < 0.001]. Thus overall, while this analysis demonstrated a familiarity effect on reducing attractiveness ratings, ratings given to the more familiar men paired with compliments were still higher than ones given to novel men not paired with compliments.

### Neuroimaging Results

**FIGURE 5:**
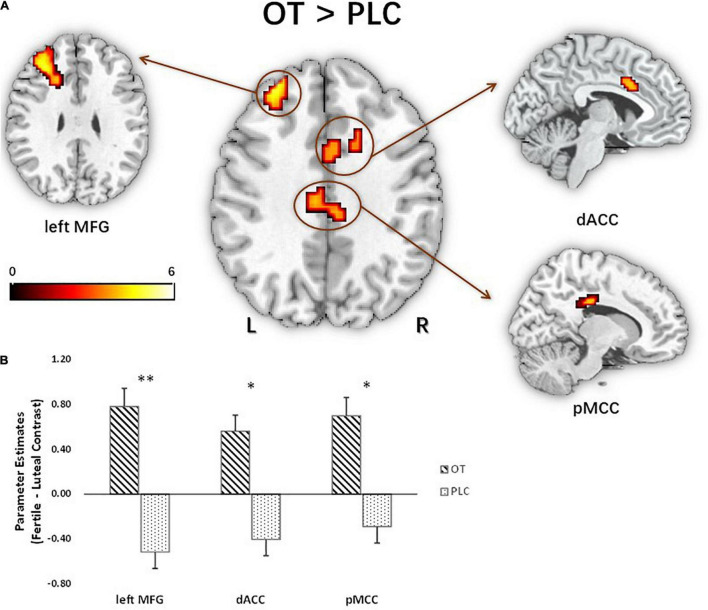
Treatment difference for novel metaphorical compliments based on cycle contrasts (fertile > luteal) in activated brain regions: left MFG, dACC, pMCC (*n* = 62). **(A)** The *t* map of the *post hoc* independent *t*-test effect showed three activated clusters peaking at the left MFG (*x* = –30, *y* = 32, *z* = 26; *t*_60_ = 5.13), dACC (*x* = 9, *y* = –28, *z* = 29; *t*_60_ = 4), and pMCC (*x* = 24, *y* = 26, *z* = 26; *t*_60_ = 4.39). Parameter estimates were extracted using the peak MNI coordinates. **(B)** Extraction based on the fertile > luteal contrast revealed that oxytocin significantly increased activation in the identified regions relative to placebo, *p < 0.05, ^**^p < 0.01, two-tailed *t*-test. Bars indicate *M* ± SE. L, left; R, right; MFG, middle frontal gyrus; dACC, dorsal anterior cingulate cortex; pMCC, posterior middle cingulate cortex.

For the fMRI analysis, we initially focused on the three-way interaction between figurativeness, cycle, and treatment, given our research interest in revealing cycle-dependent treatment effects on responses to different types of compliments. Although a significant interaction was found in the behavioral analysis indicating that women in the OT group expressed a decreased preference for novel metaphorical compliments during the fertile phase of their cycle, no corresponding significant interaction was found in the whole brain imaging analysis. Subsequent analysis of two-way interaction effects between treatment and cycle phase for each of the figurative conditions separately did, however, reveal a significant interaction effect for the faces paired with metaphorical compliments in the left middle frontal gyrus (MFG) (*F*_1_,_60_ = 26.30, *P*_FWE_ = 0.005, *k*_E_ = 156, MNI = −30/32/26). *Post hoc* examination by means of voxel-wise independent *t*-tests in SPM on the novel metaphorical compliment condition revealed that compared with the PLC group the OT group exhibited differences between the luteal and fertile phase in responses by a frontal network comprising the left MFG, *t*(60) = 5.13, *P*_FWE_ = 0.003, *k*_E_ = 209, MNI = −30/32/26; posterior middle cingulate cortex (pMCC), *t*(60) = 4.39, *P*_FWE_ = 0.037, *k*_E_ = 108, MNI = 9/−28/29; and dorsal anterior cingulate cortex (dACC), *t*(60) = 4.00, *P*_FWE_ = 0.015, *k*_E_ = 141, MNI = 24/26/26, see Figure 5A. Subsequent inspection of extracted parameter estimates from these regions revealed that the OT-treated women displayed greater responses during the fertile as compared to the luteal phase to the faces of men paired with novel metaphorical compliments, whereas the PLC-treated women displayed greater responses during their luteal as compared to the fertile phase (see Figure 5B). With Spearman analysis, the extraction of parameter estimates was correlated with attractiveness ratings on the differential contrast (fertile versus luteal phase) during processing of novel metaphorical compliments. Its result further revealed that the fertile-luteal difference in attractiveness ratings was negatively correlated with the beta value difference of the identified brain regions in the entire sample (left MFG: ρ = −0.36, *p* = 0.01; pMCC: ρ = −0.27, *p* = 0.03; dACC: ρ = −0.24, *p* = 0.06 marginal). Corresponding voxel-wise *post hoc* analyses via independent *t*-tests in SPM for the other two compliment conditions of conventional metaphors and literal expression revealed no significant results.

**FIGURE 6:**
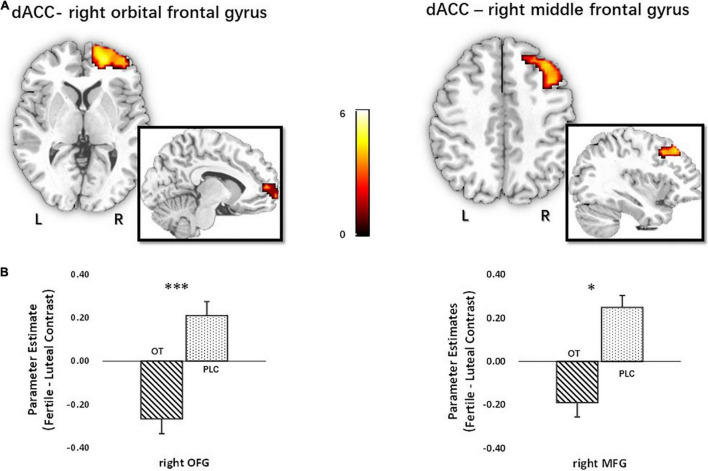
Functional connectivity analysis with dACC seed for novel metaphorical compliments based on cycle contrasts (fertile > luteal) (*n* = 62). **(A)** The *t* map of functional connectivity of dACC seed with right OFG (*x* = 21, *y* = 53, *z* = –1; *t*_60_ = 5.40) and right MFG (*x* = 39, *y* = 29, *z* = 44; *t*_60_ = 4.32). Parameter estimates were extracted using the peak MNI coordinates. **(B)** Extraction based on the fertile > luteal contrast revealed that oxytocin significantly decreased the functional connectivity of dACC seed with right OFG and right MFG relative to placebo, *p < 0.05, ^***^p < 0.001, two-tailed *t-*test. Bars indicate *M* ± SE. L, left; R, right; dACC, dorsal anterior cingulate cortex; OFG, orbital frontal gyrus; MFG, middle frontal gyrus.

To further explore whether OT changed the network-level communication of the identified regions during the processing of novel metaphorical compliments by women in their fertile phase, a functional connectivity analysis of generalized context-dependent psychophysiological interactions was employed (i.e., gPPI; McLaren et al., 2012). Based on the BOLD activation analysis results, the three identified regions (left MFG, pMCC, and dACC) were used as seeds for a seed-to-voxel gPPI analysis on the whole-brain level. The results showed that the strength of functional connectivity between dACC and right orbital frontal cortex (right OFC) [*t*(60) = 5.40, *P*_FWE_ < 0.001, *k*_E_ = 256, MNI = 21/53/−1], and right MFG [*t*(60) = 4.32, *P*_FWE_ = 0.021, *k*_E_ = 112, MNI = 39/29/44], decreased in women treated with OT relative to those treated with PLC (see Figure 6).

## Discussion

With a double-blind placebo-controlled within-subject mixed fMRI design, this study investigated the modulatory effects of intranasal OT on women’s perception of the attractiveness of men who paid verbal compliments of various topics and figurativeness during different phases of the menstrual cycle. Overall, results showed that OT particularly attenuated the increased attractiveness that women in their fertile phase usually exhibit for unfamiliar men who compliment their appearance using novel metaphorical language. This behavioral effect of OT was paralleled by women in their fertile phase showing enhanced responses to the faces of men paying such novel metaphorical compliments in the language (left MFG) and cognitive conflict processing regions (pMCC and dACC) and decreased functional connectivity between them (dACC – MFG) and between the dACC and brain reward centers (right OFG).

Contrary to our initial hypothesis, intranasal OT reduced the increased attraction women usually exhibit toward men using novel metaphorical language to compliment their appearance during the ovulatory phase of their cycle. During their ovulatory phase, women are hypothesized to have a mating strategy of seeking *good genes* in a male partner (Gangestad et al., 2004), which is evidenced by their behavioral tendencies to advertise their physical attractiveness during this period (Haselton et al., 2007), crave variety (Durante and Arsena, 2015), and show more interest in men who are more attractive than their partners (Grebe et al., 2016). Men using novel metaphorical language may be signaling greater intelligence and creativity indicative of good genes, but they may also be signaling a greater novelty seeking (Roney, 2009) and sexual interest (Roney and Simmons, 2013) indicative of a more short-term mating interest (Gangestad et al., 2004; Gao et al., 2017a). Indeed, in our current study, we have also confirmed that an independent group of female subjects did rate the novel metaphorical compliments used by men as showing greater sexual and short-term relationship interest than interest in long-term relationships and bonding.

The effect of intranasal OT in preventing the increased attractiveness of men using novel metaphorical forms of compliment at mid-cycle suggests that its actions may be antagonistic to those of the increased estrogen concentrations occurring at this time. There is evidence that estrogen can facilitate the expression of OT receptors (Bale et al., 1995). Thus the fact that OT only produced effects during the ovulatory and not the luteal phase indicates that paradoxically estrogen may both enhance women’s perception of men’s attraction who produce novel metaphorical compliments while at the same time priming the OT system to be more responsive to inhibiting this effect. In this context, however, whereas endogenous estrogen changes are physiologically regulated, those of OT should mainly be evoked by cues associated with the context of assessing the qualities of a potential mate. As such, while endogenous estrogen increases may serve to facilitate interest in potential mates showing “good gene” qualities, endogenous OT increases evoked by interactions with potential mates may serve to blunt this additional attraction to allow a greater consideration of individuals with stronger long-term bonding and parenting qualities. Interestingly, in previous research, we have shown that intranasal OT reduces single women’s interest in having short-term relationships with men who have been unfaithful in a previous relationship but increases interest in having long-term relationships with faithful ones in a previous relationship (Xu et al., 2020). This finding also suggests that OT is biasing mating preference toward men considered to have long-term bonding and parental qualities compared with ones more likely to want short-term sexual relationships.

A number of previous studies have suggested that intranasal OT may strengthen or maintain existing relationships in both monkeys (Smith et al., 2010) and humans (Scheele et al., 2012, 2013, 2015; Zheng et al., 2021). In marmosets, OT facilitated mutual social behaviors in pairs, which was inhibited by an OT receptor antagonist (Smith et al., 2010). In humans, in both sexes, intranasal OT increased the perceived attractiveness of a romantic partner and corresponding activation in brain reward regions but only for the partner and not for other familiar individuals of the opposite sex (Scheele et al., 2012, 2015). Furthermore, OT was found to influence men who were in an existing stable relationship, but not ones who were single, to keep a greater distance away from attractive unfamiliar women (Scheele et al., 2012) and to enhance the attractiveness of their female partners (Scheele et al., 2013). Additionally, OT’s effects on increasing tolerance of either imagined or simulated romantic partner infidelity in both sexes (Zheng et al., 2021) and reducing couple conflict (Ditzen et al., 2009) suggest its acting to promote maintenance of existing relationships. Interestingly, the effect of OT on increasing the attractiveness of male romantic partners for their female counterparts and corresponding increased responses in brain reward regions was reported to be abolished in those taking oral contraceptives (Scheele et al., 2016). This, therefore, further supports the possibility that the effects of oxytocin and sex hormones on attractiveness may be antagonistic to some extent.

Although some studies have reported that OT concentrations are increased in both sexes during sexual orgasm (Blaicher et al., 1999) or in response to social touch (Li et al., 2019), and may act synergistically with estrogen (Amico et al., 1981; Young et al., 1998; Patisaul et al., 2003; Salonia et al., 2005), there is still no compelling evidence that OT actually increases sexual desire in women and potentially the increased release following orgasm or in response to touch may function primarily to strengthen bonds between partners. Indeed, the study showing that OT facilitated social sharing behaviors in pair-bonded marmosets found no evidence that it increased female sexual behavior (Smith et al., 2010). However, given that there is some evidence for OT increasing estrogen and decreasing testosterone concentrations in women (Procyshyn et al., 2020) it is possible that its modulation of sex steroid hormones might have some effects on female sexual arousal.

In addition, the findings that compliments targeting landscapes are more effective than ones targeting women’s appearance is mainly driven by the selective effect of oxytocin reducing the impact of the novel compliments targeting a woman’s appearance. The very type of language that oxytocin had impact on was actually the novel metaphor targeting women’s appearance, as indicated by the *post hoc* exploration of the significant figurativeness x topic x treatment interaction. Of note, however, the scenario where men complimented landscapes was also in a flirtation context since the female subjects were told that both appearance and landscape compliments were composed by the male writers to compliment a future partner using either poetry or prose. Gersick and Kurzban (2014) pointed it out that human courtship includes a series of covert and overt behaviors, which might be from “the eloquent reverence of love poetry to the blunt aggression of catcalling.” Flirtatious language is often ambiguous in that it can communicate signals of both a friendly and sexual interest (Hall et al., 2008). Therefore, that oxytocin only reduced attractiveness ratings of novel metaphorical compliments targeting a female’s appearance may reflect the fact that they are more overtly sexual than ones targeting landscapes.

The left MFG showed increased responses to men’s faces who had produced novel metaphorical compliments under OT compared to PLC treatment. Activation of the left MFG is mainly associated with viewing written Chinese characters (Cao et al., 2017). This region is also crucial for comprehending novel metaphors (Mashal et al., 2013) due to its regulation and control of working memory in high-order cognitive processing (Brunoni and Vanderhasselt, 2014). Oxytocin has been proposed to play a significant role in modulating multimodal linguistic abilities (Theofanopoulou, 2016) and regulating the salience of social cues (Shamay-Tsoory and Abu-Akel, 2016). Hence, in our study, OT may improve the cognitive processing of Chinese metaphors used in cross-sex communication via its effects on the MFG.

Both dACC and pMCC also showed increased responses to the faces of men who had produced novel metaphorical compliments. The dACC is considered an essential region in the control of decision-making, particularly in cognitive and emotional conflict (Heilbronner and Hayden, 2016), and the MCC plays a role in monitoring decision-making (Apps et al., 2013). Mate selection is also a decision-making activity, and in the current study, female subjects were provided with both linguistic and facial cues to guide their choice of a potential mate. Additionally, the dACC is involved in regulating self-control (i.e., intentional adjustment of choice in the face of short-term gain) to make decisions with greater potential long-term benefit (Ainslie, 1975; Blanchard et al., 2015). Given that novel metaphorical compliments may sound romantic and tempting to women (Yang et al., 2019), greater dACC activation may increase women’s emotional self-control to ignore individuals who use them in favor of others whose language is more indicative of intent for a serious long-term relationship (Assadi et al., 2009). However, this possibility requires further experimental support.

The functional connectivity between dACC and both the right OFC and MFG was weakened by OT when subjects viewed the faces of men who produced novel metaphorical compliments. The OFC is linked with reward and hedonic experience (Kringelbach, 2005) and helps make decisions about the motivational value of reward outcomes (Tremblay and Schultz, 1999). Unlike the dACC, which plays a crucial role in the integration of multiple decision parameters in order to encode reward predictions, OFC is presumed to be involved in a dynamic evaluation of a current choice compared with previously experienced choices (Wallis and Kennerley, 2011), thus complementing dACC in terms of long-term decision making (Wallis, 2007). The fact that OT weakens the strength of the functional connectivity between the dACC and OFC specifically in evaluating the attractiveness of men associated with flirtatious compliments may reflect the decision that their long-term reward value is lower than individuals producing more conventional compliments. Overall, therefore, OT facilitation of dACC responses may reflect the greater conflict in decision making and weaken the perceived reward value of individuals producing flirtatious compliments more indicative of a sexual rather than bonding intent by reducing functional connectivity between dACC and OFC.

There are some limitations to this study. Firstly, other hormones that might have possibly influenced behavior (Roney, 2016) were not controlled. Studies in the future should focus on the interactive effect of multiple hormonal systems on human behaviors. Secondly, estrogen was not measured directly but inferred through indirect measurement of the half-rations urine LH detection used to determine the fertility window, although the LH level was a more objective index of ovulation than self-reported menstrual cycle dates. Thirdly, the study only focused on female subjects, and given that OT has been reported to produce several sex-dependent effects (Dumais and Veenema, 2016; Gao et al., 2016), including in the context of mate choice (Xu et al., 2020), findings in men could be different.

In summary, the current study results not only support the assumption that OT can influence the perceived attractiveness of courtship language in terms of metaphor preference related to mate choice but also suggest a potential neural and hormonal mechanism. Thus, increased OT in women at the fertile phase during interactions with potential partners may help to promote the choice of individuals more likely to offer long-term bonds and relationships by a combination of enhancing activity in cognitive decision making regions (MFG, dACC, and pMCC) while simultaneously weakening the reward value of individuals offering more short-term sexual relationships (weakened dACC – OFC functional connectivity).

## Data Availability Statement

The datasets presented in this study can be found in online repositories. The names of the repository/repositories and accession number(s) can be found below: https://osf.io/qgu3x/?view_only=74ee6a1c4ed04b639845fd4b82ffbbfb.

## Ethics Statement

The studies involving human participants were reviewed and approved by the Local Ethics Committee of the University of Electronic Science and Technology of China. The patients/participants provided their written informed consent to participate in this study. Written informed consent was obtained from the individual(s) for the publication of any potentially identifiable images or data included in this article.

## Author Contributions

ZG and KK designed the research. ZG, XM, and FX performed the research. ZG, XZ, BB, and JK analyzed the data. ZG, KK, BB, and SG wrote the manuscript. All authors contributed to the article and approved the submitted version.

## Conflict of Interest

The authors declare that the research was conducted in the absence of any commercial or financial relationships that could be construed as a potential conflict of interest.

## Publisher’s Note

All claims expressed in this article are solely those of the authors and do not necessarily represent those of their affiliated organizations, or those of the publisher, the editors and the reviewers. Any product that may be evaluated in this article, or claim that may be made by its manufacturer, is not guaranteed or endorsed by the publisher.

## Footnotes

1
https://clinicaltrials.gov/show/NCT03144115

2
http://ccl.pku.edu.cn:8080/ccl_corpus

3
http://www.fil.ion.ucl.ac.uk/spm/spm8
