# Supplementary material for: Oxytocin Reduces the Attractiveness of Silver-Tongued Men for Women During Mid-Cycle
Source: Front Neurosci. 2022 Apr 28;16:760695. doi: 10.3389/fnins.2022.760695 (PMC9097854; doi:10.3389/fnins.2022.760695)
Supplement: Supplementary file 1 [file Data_Sheet_1.docx]

**Oxytocin reduces the attractiveness of silver-tongued men for women during mid-cycle**


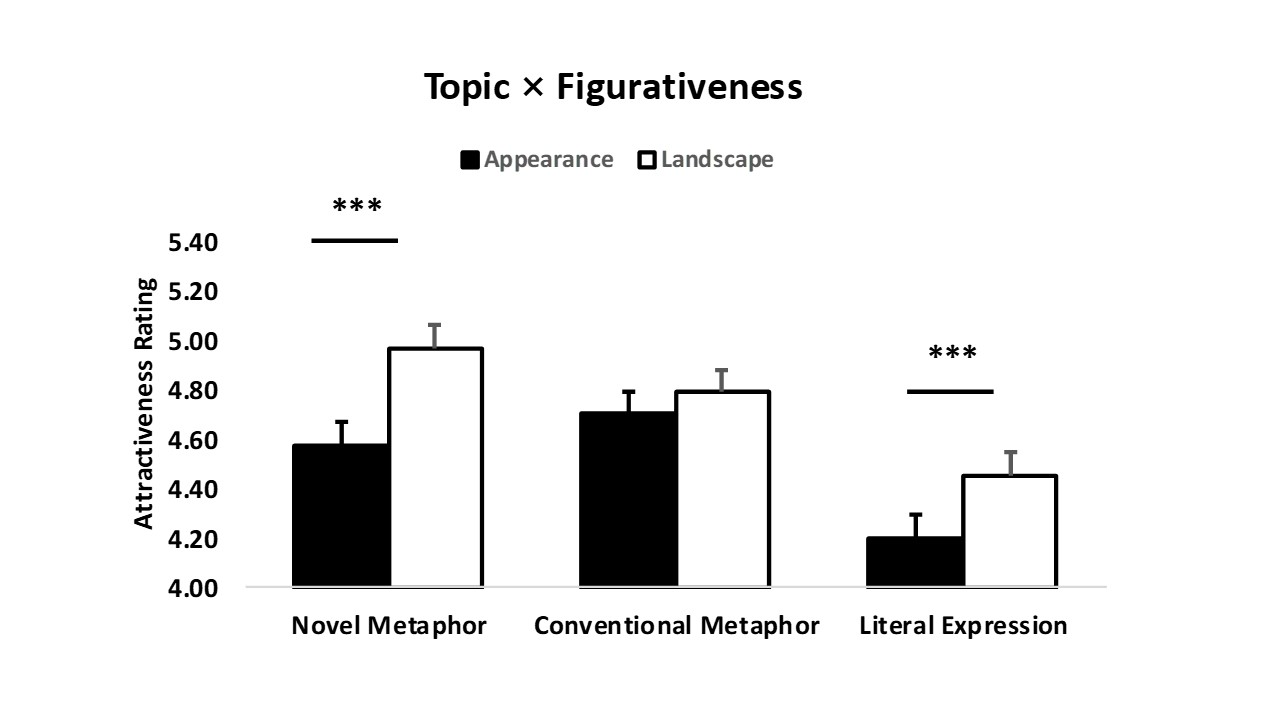


Figure S1. Figurativeness × topic interaction effect on attractiveness ratings independent of menstrual cycle and treatment (n=62). ****p* < 0.001, two-tailed *t* test. Bars indicate *M* ± SE.


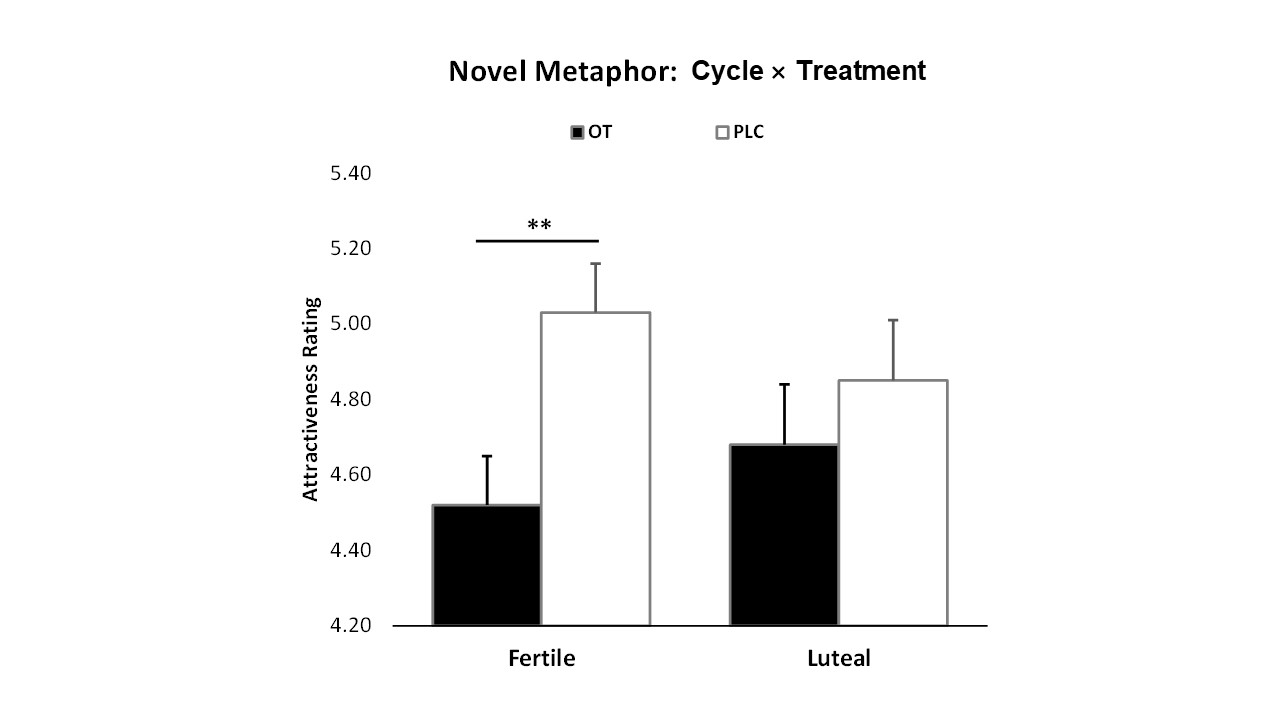


Figure S2. Cycle × treatment interaction effect only found in condition of novel metaphorical compliments (n=62). ***p* < 0.01, two-tailed *t* test. Bars indicate *M* ± SE.

| Table S1. Ratings of six sentence types in terms of six criteria. | | | | | | | | | | | | | | |  |
| --- | --- | --- | --- | --- | --- | --- | --- | --- | --- | --- | --- | --- | --- | --- | --- |
| Criteria | | Sentence Type | | | N | | M | | SD | | *F* & *p* | | Bonferroni ^a^ | | |
| figurativeness | | | 1-nma^a^ | | 48 | | 5.15 | | 0.32 | | *F* = 935.368 *p* < 0.001  *η*2 p = 0.943 | | 1/2/4/5>6>3 | | |
|  |  |  | 2-cma | | 48 | | 5.10 | | 0.31 | |  |  |  |  |  |
|  |  |  | 3-lea | | 48 | | 2.08 | | 0.32 | |  |  |  |  |  |
|  |  |  | 4-nml | | 48 | | 5.14 | | 0.37 | |  |  |  |  |  |
|  |  |  | 5-cml | | 48 | | 4.99 | | 0.36 | |  |  |  |  |  |
|  |  |  | 6-lel | | 48 | | 2.30 | | 0.36 | |  |  |  |  |  |
|  | | | | | | | | | | | | | | |  |
| familiarity | | | 1-nma | | 48 | | 3.41 | | 0.24 | | *F* = 316.119  *p* < 0.001  *η*2 p = 0.849 | | 3/6>2/5>1>4 | | |
|  |  |  | 2-cma | | 48 | | 4.83 | | 0.35 | |  |  |  |  |  |
|  |  |  | 3-lea | | 48 | | 5.22 | | 0.43 | |  |  |  |  |  |
|  |  |  | 4-nml | | 48 | | 3.16 | | 0.35 | |  |  |  |  |  |
|  |  |  | 5-cml | | 48 | | 4.70 | | 0.29 | |  |  |  |  |  |
|  |  |  | 6-lel | | 48 | | 5.18 | | 0.41 | |  |  |  |  |  |
|  | | | | | | | | | | | | | | |  |
| appropriateness | | | 1-nma | | 48 | | 4.58 | | 0.37 | | *F* = 35.571 *p* < 0.001  *η*2 p = 0.387 | | 2/3/5/6>1/4 | | |
|  |  |  | 2-cma | | 48 | | 5.16 | | 0.35 | |  |  |  |  |  |
|  |  |  | 3-lea | | 48 | | 5.06 | | 0.37 | |  |  |  |  |  |
|  |  |  | 4-nml | | 48 | | 4.49 | | 0.30 | |  |  |  |  |  |
|  |  |  | 5-cml | | 48 | | 5.09 | | 0.27 | |  |  |  |  |  |
|  |  |  | 6-lel | | 48 | | 5.02 | | 0.33 | |  |  |  |  |  |
|  | | | | | | | | | | | | | | |  |
| valence | | | 1-nma | | 48 | | 5.07 | | 0.32 | | *F* = 1.991 *p* = 0.080  *η*2 p = 0.034 | |  | | |
|  |  |  | 2-cma | | 48 | | 5.05 | | 0.28 | |  |  |  |  |  |
|  |  |  | 3-lea | | 48 | | 5.12 | | 0.30 | |  |  |  |  |  |
|  |  |  | 4-nml | | 48 | | 4.98 | | 0.27 | |  |  |  |  |  |
|  |  |  | 5-cml | | 48 | | 4.97 | | 0.25 | |  |  |  |  |  |
|  |  |  | 6-lel | | 48 | | 5.06 | | 0.21 | |  |  |  |  |  |
|  | | | | | | | | | | | | | | |  |
| imageability | | | 1-nma | | 48 | | 4.20 | | 0.48 | | *F* = 83.952 *p* < 0.001  *η*2 p = 0.58 | | 1/2/4/5>3/6  4>2/5 | | |
|  |  |  | 2-cma | | 48 | | 3.79 | | 0.42 | |  |  |  |  |  |
|  |  |  | 3-lea | | 48 | | 2.73 | | 0.54 | |  |  |  |  |  |
|  |  |  | 4-nml | | 48 | | 4.36 | | 0.67 | |  |  |  |  |  |
|  |  |  | 5-cml | | 48 | | 3.54 | | 0.34 | |  |  |  |  |  |
|  |  |  | 6-lel | | 48 | | 2.90 | | 0.50 | |  |  |  |  |  |
|  | | | | | | | | | | | | | | |  |
| arousal | | | 1-nma | | 48 | | 4.79 | | 0.54 | | *F* = 60.341 *p* < 0.001  *η*2 p = 0.517 | | 1/2/4/5>3/6  1/2>5 |  |  |
|  |  |  | 2-cma | | 48 | | 4.74 | | 0.46 | |  |  |  |  |  |
|  |  |  | 3-lea | | 48 | | 3.94 | | 0.32 | |  |  |  |  |  |
|  |  |  | 4-nml | | 48 | | 4.56 | | 0.54 | |  |  |  |  |  |
|  |  |  | 5-cml | | 48 | | 4.31 | | 0.38 | |  |  |  |  |  |
|  |  |  | 6-lel | | 48 | | 3.57 | | 0.26 | |  |  |  |  |  |
| 1-nma=novel metaphor targeting appearance, 2-cma=conventional metaphor targeting appearance, 3-lea=literal expression targeting appearance, 4-nml=novel metaphor targeting landscape, 5-cml=conventional metaphor targeting landscape, 6-lel=literal expression targeting landscape. a. *p* < 0.05 | | | | | | | | | | | | | | |  |
